# Supplementation of caffeine and sodium bicarbonate together could not improve performance and performance-related factors in CrossFit participants: a randomized, double-blind, placebo-controlled study

**DOI:** 10.1080/15502783.2023.2206390

**Published:** 2023-05-08

**Authors:** Amirhosein Ziyaiyan, Fatemeh Shabkhiz, Martin Hofmeister

**Affiliations:** a Department of Exercise Physiology, Faculty of Physical Education and Sports Science, University of Tehran, Tehran, Iran; b Department of Exercise Physiology, Faculty of Physical Education and Sports Sciences, University of Tehran, Tehran, Iran; c Department Food and Nutrition, Consumer Centre of the German Federal State of Bavaria, Munich, Germany

**Keywords:** Sports performance, muscle strength, sports nutrition, supplementation

## Abstract

**Background:**

CrossFit includes weightlifting, powerlifting, and gymnastics in various combinations of overloads and repetitions with limited rest periods or no rest between training sets. Due to the novelty of CrossFit, there are few studies on the effect of nutritional strategies on the acute response to this type of sports activity. This study examined the effect of caffeine (CAF) and sodium bicarbonate (NaHCO_3_) ingestion separately and in combination on the performance and rate of perceived exertion (RPE) during the Cindy CrossFit workout (Cindy) in CrossFit participants.

**Method:**

In a double-blind, crossover, randomized, placebo-controlled trial, 20 CrossFit participants underwent five experimental conditions, including control (CON), placebo (PLA), CAF, NaHCO_3_, and CAF + NaHCO_3_ (7 days to wash-out between assessment sessions) before completing the Cindy protocol (age: 22.30 ± 2.88 years, body mass index: 25.22 ± 2.51 kg/m^2^). Capsules containing 6 mg/kg body weight (BW) CAF were consumed 50 min before the Cindy workout while 0.3 g/kg BW NaHCO_3_ was consumed for 3 days, leading to 120, 90, and 60 min before the Cindy workout. Performance, RPE, muscular power (MP), handgrip strength (HGS), and maximum heart rate (MHR) were measured before and shortly after the Cindy.

**Results:**

The performance of CrossFit participants during the Cindy protocol was not significantly improved following CAF, NaHCO_3_, and CAF + NaHCO_3_ (*P* > 0.05). In contrast, RPE during and at the end of the Cindy was significantly decreased following CAF + NaHCO_3_ consumption compared to PLA and CON (*P* = 0.001, *P* = 0.02). However, MP (*P* = 0.82) and HGS (*P* = 0.52) were not significantly different between conditions. Also, MHR was significantly greater following CAF, NaHCO_3_, and CAF + NaHCO_3_ consumption than CON (*P* = 0.01).

**Conclusion:**

CAF + NaHCO_3_ supplementation decreased RPE despite significantly increased MHR, but with no significant effect on performance, HGS, or MP. Therefore, CrossFit participants may benefit from the ergogenic effects of CAF and NaHCO_3_ when consumed separately or together.

## 1. Background

CrossFit includes Olympic weightlifting, powerlifting, and gymnastics in various combinations of overloads and repetitions with limited rest periods or no rest between training sets. Therefore, CrossFit combines intense and resistance training [1].

High-intensity exercise leads to an accumulation of lactate and hydrogen ions (H^+^) as a byproduct of the anaerobic glycolysis pathway because of the limited oxygen availability caused by the increased rate of glycolysis in active muscle cells [2]. Cellular acidosis decreases energy production by lowering the proton gradient between the mitochondrial matrix and the cell cytoplasm. One of the most potent causes of perceived fatigue [3] is increased intracellular acidosis, caused by the inhibition of essential enzymes in energy metabolism [4] as well as reduced muscle stimulation [5]. Maintaining muscular contraction depends on buffer systems that remove H^+^ from muscle cells. Muscle acidity is controlled by intracellular, extracellular, and dynamic buffers during high-intensity exercise [6], and bicarbonate (HCO_3_-), in particular, is crucial to the blood buffering system [7]. Theoretically, performance under high-intensity conditions may be enhanced by raising the HCO_3_ concentration. As a result, supplementation with sodium bicarbonate (SB) has been suggested as an ergogenic supplement [8]. Several studies have demonstrated that oral SB supplementation enhances blood’s pH and HCO_3_ content [9]. Increasing glycogenolysis activity and dependence on muscle glycogen reserves as energy sources during exercise have been linked to metabolic alkalosis in skeletal muscle [10].

One of the most popular supplements among athletes is caffeine (CAF; 1,3,7-trimethylxanthine). Through its mechanism of action, which includes effects on the central nervous system (CNS), an increase in the amount of free fatty acids that are readily available, and a direct effect on muscle contraction, CAF supplementation at doses of 3–6 mg/kg body weight (BW) improves long-term aerobic exercise performance [11]. Several studies have demonstrated that CAF intake decreases extracellular potassium concentration during high-intensity and short-term exercise while increasing plasma catecholamine concentration, postponing exhaustion, and enhancing performance [12].

There are few studies about the effect of nutritional strategies on the acute response to high-intensity exercise [13]. CAF is a popular supplement among athletes used as an ergogenic aid to improve physical function, delay fatigue, and increase muscle strength and power [14]. One possible mechanism of CAF is its ability to act as an adenosine antagonist. When CAF binds to adenosine receptors, it can increase alertness [15] and reduce the CNS’s perception of pain, fatigue, and effort [16]. In addition, CAF stimulates the release of beta-endorphins, which helps increase pain and stress tolerance [11]. In a cross-sectional study by Green et al., they found that CAF could increase the number of repetitions in a high-intensity resistance exercise [17]. CAF may also play a role in increasing muscle strength. However, the mechanisms are still unknown [17]. In a study, Drum et al. [18] showed that CrossFit participants experienced a higher rate of perceived exertion (RPE) than those who exercised according to the guidelines of the American College of Sports Medicine. Several studies have examined the effect of CAF supplementation [14,19] and SB supplementation [9] on the performance, strength, and power of CrossFit participants. Also, in a study conducted by Rezaei et al. [20], the effect of CAF and SB consumption on the performance, strength, and perceived exertion of professional karate participants was examined.

Consumption of NaHCO_3_ with CAF is likely to be a beneficial additive for athletes involved in high-intensity exercise [21]. However, the combination of these two supplements has not been directly studied in CrossFit sports. Therefore, this study will examine the effect of CAF and SB supplementation alone and together on CrossFit performance and RPE in CrossFit participants.

## 2. Methods

### 2.1. Participants

All male members of the Koohpaye Box club were invited to participate in this study. The Koohpaye Box club is one of the top CrossFit gyms in Tehran City, Iran. This study was conducted in one CrossFit club to eliminate the potential effects of variations in training programs between different clubs. CrossFit participants were included in this study if they had at least 2 years of experience in CrossFit, did not consume any supplements 3 months before and during the study, and were not heavy CAF users (CAF ≤125 mg/d). A total of 20 male CrossFit participants (age: 22.30 ± 2.88 years; height: 1.78 ± 6.84 m; body mass: 80.6 ± 12.05 kg) completed the study. To determine whether the number of participants was adequate for this study, we used a priori power analysis using the G*Power 3.1.9.2 [22]. The study was conducted throughout the annual training program’s 5-week preparation phase. During the preparation phase, CrossFit participants trained five sessions per week throughout the transition phase, including five CrossFit-specific training sessions that included strength, endurance, and power training.

In the first visit, participants’ height and body mass were measured using a digital stationary stadiometer SECA 264 (Hamburg, Germany) and a calibrated digital floor scale SECA 874 (Hamburg, Germany), respectively.

The Human Ethics Research Committee approved this study of the Sport Sciences Research Institute of Iran according to the compliance with the Ethical Standards in Research of the Ministry of Science, Research and Technology, with the code IR.SSRI.REC.1400.1276 (date of registration: 23/11/2021), as well as operating in accordance with the Declaration of Helsinki. Before participating in the study, CrossFit participants signed informed consent.

### 2.2. Experimental design

**Figure 1.**
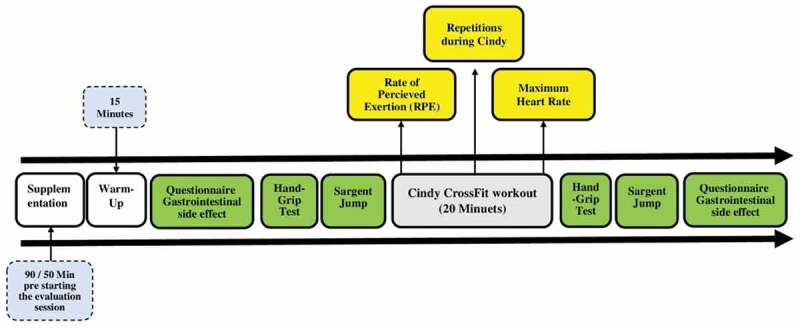
Schematics of experimental steps.

The current investigation was a double-blind, crossover, randomized, placebo-controlled trial. To ensure that both researchers and participants were blind to the conditions, an independent pharmacist prepared and administered all supplements. Following two sessions of protocol familiarization, participants were randomly assigned to one of five conditions: CAF, NaHCO_3_, a combination of CAF and NaHCO_3_, placebo (PLA), and control (CON). Each participant was randomly assigned to these conditions to control for the possible effects of training factors during the research period. The CON session data were used for comparative analysis. The washout period was 7 days, and all evaluations were performed simultaneously (between 09:00 AM and 12:00 PM) to account for circadian changes. During each evaluation session, performance was measured by protocol; RPE and heart rate were measured during protocol, and the handgrip strength (HGS) and muscular power (MP) were measured immediately before and immediately after protocol to measure the effect of supplementation on these two factors in the fatigue period caused by the implementation of the protocol. The schematics of the experimental steps are shown in Figure 1.

### 2.3. Supplementation protocol

The Iran Gelatin Capsule Co. packaged the supplements and PLA (cellulose) in identical gelatin capsules, and participants could not determine the contents of the capsules. Supplementation began 3 days before each protocol session, with NaHCO_3_ (0.3 g/kg BW/d, Bio-Tech Industries Co., Hungary) or PLA taken with breakfast, lunch, and supper. The participants drank plenty of water (250–300 ml) with capsules to help prevent any gastrointestinal (GI) discomfort and to aid in the absorption of supplements. This loading method is chosen since it has been demonstrated to minimize GI discomfort and maintain blood carbonate levels 1 day after NaHCO_3_ consumption [23]. On the evaluation day, capsules containing NaHCO_3_ (0.1 g/kg BW) or PLA were administered 120, 90, and 60 min before protocol, and capsule containing either CAF 6 mg/kg BW (caffeine anhydrous, Biotech industries Co., Hungary) or PLA was ingested 50 min before protocol [24].

### 2.4. Dietary control

Participants were informed to refrain from eating any beverages or meals containing baking soda, CAF, or alcohol throughout the experiment and doing high-intensity activity within 24 h before protocol. Participants were given a list of typical foods and beverages that were safe to consume and those that should be avoided. Although participants did not record their dietary intake during the experiment, they were recommended to record it 24 h before the first condition to replicate it before the subsequent sessions. Participants consumed a standardized snack (white bread and boiled eggs) containing 1.5 g/kg BW carbs, 20 g protein, and 10 g fat 150 min before each protocol to reduce the possibility of GI distress.

### 2.5. Performance, HGS, and SJ

#### 2.5.1. Cindy CrossFit workout protocol

In this study, the Cindy CrossFit workout protocol was chosen because it is a standard workout in CrossFit and has been used in previous studies [18]. This protocol was used to evaluate the performance of participants. This protocol includes 5 pull-ups, 10 push-ups, and 15 air squats, which one should try to perform as many repetitions as possible by performing the exercises correctly in 20 min. During the execution of the movements, the official and qualified referee of CrossFit Level 1 evaluated the performance of the participants and in case of incorrect execution of the exercises, the counting was not done and it was warned verbally. The protocol was performed indoors in a gym, with the temperature set to 21–24°C. Each participant performed the workout alone, with no timing device visible to them and without music.

#### 2.5.2. Handgrip strength

A handgrip dynamometer was used to assess hand muscle strength. After warming up, the participants held the handgrip dynamometer (Takei Hand Grip Strength Digital Dynamometer, Japan) with the dominant hand in front of the body, with the elbow fully extended, and pressed the handgrip dynamometer with maximum force once with a single push. Each participant repeated the test three times with 30-s rest, and the best record was considered the primary record [25]. The handgrip test was performed immediately before and after the Cindy protocol with the dominant hand.

#### 2.5.3. Sargent jump

The Sargent jump test was used to assess the power of lower body muscles. After warming up, the participants stood side to a wall and reached up with the hand closest to the wall. The point of the fingers was marked by keeping the feet flat on the ground. The participant then stood away from the wall and jumped as high as possible using both arms and legs. The highest touchpoint was identified. The difference between the highest touchpoint and the standing reach point was used to calculate the jumping score. Each participant performed the jumping three times with a 30-s interval rest, and the best record was considered the primary record [26]. Participants performed three Sargent jumps immediately before and after the pre-and-post HGS.

### 2.6. Maximum heart rate and RPE measurement

A Polar heart rate monitor recorded the heart rate during the Cindy protocol (Polar, V800, H10 heart rate sensor, Electro, Oy, Kempele, Finland). In addition, after every 4 min and at the end of the Cindy protocol, the RPE score was recorded on a scale of 1–10.

### 2.7. Gastrointestinal questionnaire

To assess the symptoms of GI discomfort, a GI questionnaire was used [27]. Participants chose numbers between 0 and 9, with 0 indicating “no problem at all” and 9 indicating “worst it has ever been.” When the score was equal to or greater than 5, the symptoms were considered severe.

### 2.8. Monitoring fatigue and training status

Coaches were asked to maintain training volume and intensity throughout the research to reduce the effect of training volume and avoid overtraining. Before each of the Cindy protocol, the well-being Hooper index questionnaire [28] was utilized to monitor and measure recovery and accumulated weariness.

### 2.9. Data analysis

Statistical analyses were conducted using a statistical software package (IBM SPSS Statistics 23.0, Armonk, NY: IBM Corp.) and were presented in mean and standard deviation (SD). After normality (i.e. Shapiro–Wilk) and variance assurance (i.e. Levene), a one-way repeated measure analysis of variance was used to compare the effect of different supplementations on performance, and maximum heart rate (MHR), HGS, and MP after each of the Cindy protocol. When the results revealed a significant difference between conditions, a Bonferroni post hoc analysis was conducted to identify the differences. To compare the RPE between different levels of the Cindy protocol, the Kruskal–Wallis test was used.

## 3. Results

### 3.1. Performance, MHR, HGS, and MP

**Figure 2.**
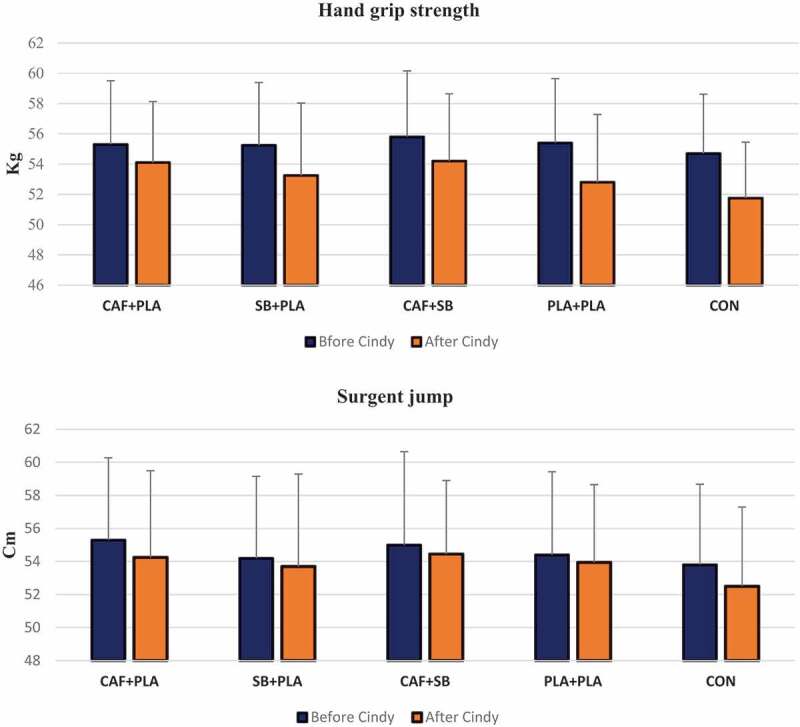
A comparison of variables between supplementations, PLA, and CON conditions before and after the Cindy protocol.

**Figure 3.**
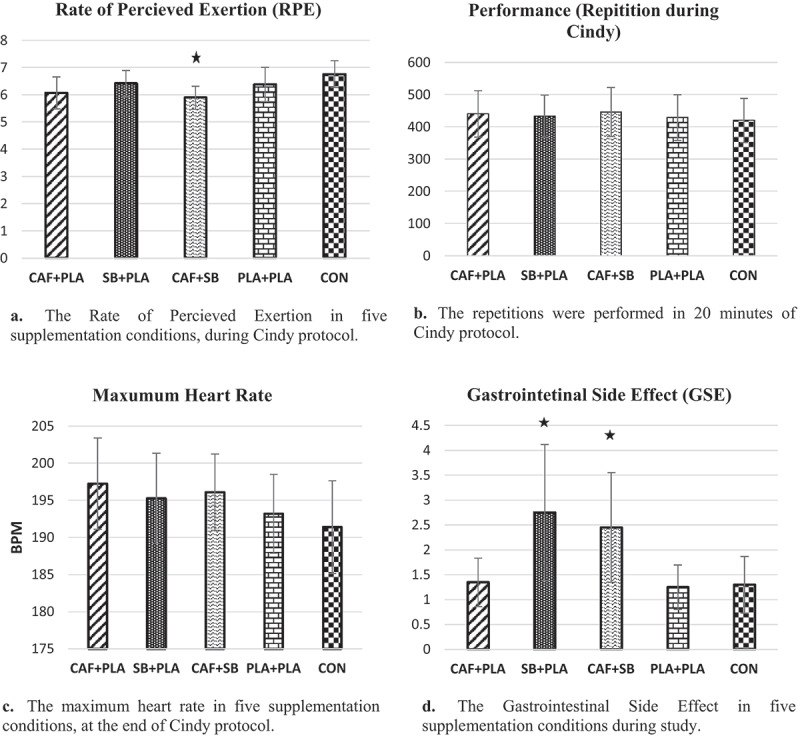
A comparison of variables between supplementations, PLA, and CON conditions during the Cindy protocol and the study.

The results did not show a significant effect of supplementation on performance in CrossFit participants during the Cindy protocol (Table 1), *F* [4, 95] = 0.40, *P *= 0.80, $\eta_{P}^{2}$ = 0.01. The MHR at the end of the Cindy protocol, as demonstrated in Figure 3, was significantly different between conditions *F*[1, 95] = 3.62,*P *< 0.001, $\eta_{P}^{2}$ = 0.13. Pairwise comparison revealed that MHR was significantly greater in CAF (197 ± 6.15 Rpm, *P* = 0.01) and CAF + NaHCO_3_ (196 ± 5.14 Rpm, *P* = 0.04) compared to CON (191 ± 6.23 Rpm). Non-significant effect of supplements was also observed on HGS *F* [1, 95] = 0.52, *P *= 0.71, $\eta_{P}^{2}$ = 0.02 and on MP *F* [1, 95] = 0.37, *P *= 0.82, $\eta_{P}^{2}$ = 0.01 following the Cindy protocol (Figure 2).

**Table 1.** Descriptive statistics of handgrip strength, lower torso muscle power, performance, maximal heart rate, rate of perceived exertion, and gastrointestinal side effects variables in five supplementation conditions (mean ± SD).

Condition	Stage	Handgrip*	Sargent jump*	Repetitions of Cindy protocol	Maximum heart rate	RPE	GSE
CAF+PLA	1	4.20 ± 55.30	4.97 ± 55.30	71.59 ± 439.60	6.15 ± 197.25	0.58 ± 6.06	0.48 ± 1.35
	2	4.02 ± 54.10	5.24 ± 54.25	—	—	—	—
NaHCO_3_+PLA	1	4.14 ± 55.25	4.94 ± 54.20	65.56 ± 432.95	6.09 ± 195.25	0.48 ± 6.41	1.37 ± 2.75
	2	4.77 ± 53.25	5.59 ± 53.70	—	—	—	—
CAF+NaHCO_3_	1	4.36 ± 55.80	5.64 ± 55.00	75.96 ± 445.35	5.14 ± 196.10	0.42 ± 5.89	1.09 ± 2.45
	2	4.44 ± 54.20	4.45 ± 54.45	—	—	—	—
PLA+PLA	1	4.24 ± 55.40	5.03 ± 54.40	70.42 ± 428.20	5.32 ± 193.20	0.63 ± 6.37	0.44 ± 1.25
	2	4.47 ± 52.80	4.69 ± 53.95	—	—	—	—
CON*	1	3.92 ± 54.70	4.87 ± 53.80	68.34 ± 419.50	6.23 ± 191.40	0.48 ± 6.75	0.57 ± 1.30
	2	3.69 ± 51.57	4.79 ± 52.50	—	—	—	—

Note: *Control condition: The subjects did not receive any supplements in this condition. *Handgrip strength and surgent jump height were measured in two stages before and after the Cindy protocol: the first time (stage 1) to evaluate the effect of supplementation in each condition on strength and power in rest condition and the second time (step 2) to evaluate the effect of supplementation on strength and power in fatigue condition. CAF, caffeine; NaHCO_3_, sodium bicarbonate; PLA, placebo; CON, control; RPE, rate of perceived exertion; GSE, gastrointestinal side effects.

### 3.2. Rate of perceived exertionE

The effects of different supplements on RPE in CrossFit participants during the Cindy protocol (Table 2) were significant, *H* [3] = 32.08, *P *≤ 0.05. Pairwise comparison revealed that RPE was significantly less in CAF (6.06 ± 0.58, *P* = 0.05) compared to NaHCO_3_ (6.41 ± 0.48) and CON (6.75 ± 0.48). A significant difference was also observed in RPE between NaHCO_3_ (6.41 ± 0.48, *P* = 0.001), CAF + NaHCO_3_ (5.89 ± 0.42), and CON (6.75 ± 0.48). Likewise, RPE was significantly less in CAF + NaHCO_3_ (5.89 ± 0.42, *P* = 0.001, *P* = 0.02) compared to PLA (6.37 ± 0.63) and CON (6.75 ± 0.48) (Figure 3a).

**Table 2.** RPE in each level of the Cindy protocol (every 4 min).

Conditions	Sig	4 min (Level 1)	8 min (Level 2)	12 min (Level 3)	16 min (Level 4)	20 min (Level 5)
CAF	0.13	2.05 ± 0.66	3.90 ± 0.83	6.30 ± 0.71	8.45 ± 0.80	9.60 ± 0.48
NaHCO_3_	0.09	2.45 ± 0.66	4.35 ± 0.79	6.50 ± 0.59	9.05 ± 0.66	9.70 ± 0.45
CAF+NaHCO_3_	0.02*	2.15 ± 0.57	4.05 ± 0.73	6.05 ± 0.73	7.95 ± 0.80	9.25 ± 0.43
PLA	0.12	2.25 ± 0.62	4.30 ± 1.05	6.75 ± 0.88	9.00 ± 0.63	9.55 ± 0.49
CON	0.18	2.50 ± 0.59	4.80 ± 0.92	7.25 ± 0.76	9.35 ± 0.57	9.85 ± 0.35

Note: CAF, caffeine; NaHCO_3_, sodium bicarbonate; PLA, placebo; CON, control.

### 3.3. Gastrointestinal symptoms

The results showed significant GI side effects with the combination of CAF and NaHCO_3_ and NaHCO separately compared to the PLA group, but these complications were temporary, were not severe, and did not affect participants’ performance. Hooper total score was similar between conditions before the Cindy protocol.

## 4. Discussion

This study investigated the ergogenic effects of CAF and NaHCO_3_ supplementation when consumed together or separately on performance, MP, HGS, MHR, and RPE before, during, and after the Cindy protocol. We hypothesized that co-ingestion of CAF and NaHCO_3_ would have a greater effect on athletic performance than CAF and NaHCO_3_ alone. However, the findings rejected the primary hypothesis and showed no additional benefits of co-ingesting CAF and NaHCO_3_ compared to CAF or NaHCO_3_.

Performance in this study was defined as the repetition of performing all exercises in each round until voluntary exhaustion. Our results indicated that all conditions improved the performance compared to PLA during the Cindy protocol. Although the differences between conditions were not statistically significant, a close review of the results showed a greater improvement in performance in CAF + NaHCO_3_ (4.0%) than CAF (2.6%) or NaHCO_3_ (1.1%) when compared to PLA. This result is in contrast with Pruscino et al. [29], Christensen et al. [30], Carr et al. [30], and Felippe et al. [31]. Pruscino et al. [29] showed an increase in performance in two sets of 200 m freestyle (30-min intervals). The results showed an increase in performance when NaHCO_3_ was consumed separately or with CAF [29]. In other studies, Carr et al. [30] and Christensen et al. [21] found higher performance in elite rowers (on a rowing ergometer) after consuming CAF separately (not with SB). It has been suggested that CAF may be a stronger ergogenic aid and that its combination with SB may balance its ergogenic effects [21,30]. In another study, Felippe et al. [31] concluded that by consuming CAF and SB together, overall performance during a single bout of judo activity (number of throws) improved significantly more than when consumed separately. These results suggest that the combination and separate effects of CAF and SB may depend on the type of exercise task [31]. Toledo et al. [32] conducted a study to investigate the acute effect of NaHCO_3_ supplementation on participants’ performance during CrossFit training. The results were consistent with our findings. The results showed that acute SB supplementation did not improve the performance of professional CrossFit participants during the Cindy protocol [32]. Also, our results follow Stein et al. [19], who showed that the performance of CrossFit participants during CAF conditions was not significantly different from that of a PLA, and that the effect of an ergogenic effect of CAF was not observed during the Cindy protocol [19].

The results of the present study showed that CAF and NaHCO_3_ supplementation, separately or in combination, did not significantly improve HGS of CrossFit participants. There was no difference between the supplementation conditions compared to PLA and CON. Also, comparing the handgrip test scores in all conditions after the Cindy protocol compared to before indicated that CAF and NaHCO_3_ supplementation separately and in combination did not affect HGS in fatigue period caused by the implementation of the Cindy protocol in each condition (Figure 2). Our results are similar to those of Mora-Rodríguez et al., who reported no difference in CAF compared to PLA conditions in the isometric strength of the handgrip after the neuromuscular test battery [33]. Also, San Juan et al., in a study aimed at evaluating CAF supplementation to improve anaerobic performance and neuromuscular activity and fatigue in Olympic boxers, did not observe a significant difference between the CAF supplementation and PLA conditions [34]. However, other studies have found improvements in HGS after CAF supplementation [35–38]. The lack of a significant effect of CAF consumption on isometric strength and the inconsistency observed in the literature may indicate that CAF supplementation is more effective on dynamic strength. Moreover, it should be noted that CAF consumption causes a greater increase in the lower body compared to the upper body functional strength [39]. Also, Ansdell et al. did not observe improvement in muscle strength after consuming 0.4 g/kg BW NaHCO_3_ 90 and 60 min before exercise in basketball players [40]. However, Coombes et al. reported that consumption of 0.3 g/kg NaHCO_3_ increased the maximum knee extension torque by up to 8% in nine male physical education students [41].

The results of the present study showed that CAF and NaHCO_3_ supplementation, separately or in combination, did not significantly improve the power of lower body muscles in CrossFit participants (Figure 3b). There was no significant difference in Sargent jump scores between supplementation conditions in comparison to PLA and CON. Also, the comparison of Sargent jump scores in each condition, before and after the Cindy protocol, indicates that CAF and NaHCO_3_ supplementation separately and together did not improve lower body power output in a fatigued state while performing the protocol in each condition (Figure 2). Mohammadi Gajvati et al., contrary to our findings, observed an improvement in the mean power and peak power in athlete subjects after NaHCO_3_ supplementation [42], while our findings are consistent with the results of Joyce et al. [43], Durkalec-Michalski et al. [44], and Zabala et al. [45, who did not observe the effect of acute NaHCO_3_ supplementation on the peak power. In a crossover study, Zabala et al. [45 investigated the effect of NaHCO_3_ supplementation 90 min before the activity in nine elite cyclists. They concluded that there is a significant effect of NaHCO_3_ supplementation on maintaining and even increasing buffering capacity but did not observe any effect on improving the peak power of participants [45]. In following our results, Joyce et al., with NaHCO_3_ supplementation 120–90 min before the test in eight elite male swimmers, concluded that NaHCO_3_ supplementation did not increase the peak power of participants [43]. One possible reason for the ineffectiveness of NaHCO_3_ supplementation on muscle power may be due to the time of consumption of supplementation [43]. Some studies have shown that the effectiveness of NaHCO_3_ supplementation decreases 60 min after ingestion [46–48]; this may be one of the possible reasons why NaHCO_3_ supplementation does not affect the Sargent jump scores in CrossFit participants (Figure 2). Also, contrary to our findings, sugar-free energy drinks containing 3 mg/kg BW of CAF increased jump height by 3.2% and muscle power by 3.8% in semiprofessional soccer players [49]. In young, high-level basketball players, despite a change in the output power of the leg muscles, a 2.1% improvement in vertical jump height was observed [50]. CAF consumption increased muscle power by 8.9% in elite female rugby players during a 15-s rebound jumping [51]. Similarly, drinks containing high doses of CAF (6 mg/kg) resulted in a 2.7% increase in vertical jump height and lower limb muscle power in soccer players [52].

Our findings showed that the combination of CAF and NaHCO_3_ significantly reduced the RPE compared to the CON and PLA conditions during the Cindy protocol (Figure 3a). The mechanism by which the combination of NaHCO_3_ and CAF reduces the RPE for a given effort is not fully understood. Still, it may result in the total effects of CAF and NaHCO_3_ on the CNS [41]. CAF acts as an adenosine antagonist in the CNS, a neurotransmitter responsible for pain and drowsiness [53]. On the other hand, extracellular H^+^ accumulation due to high-intensity exercises activates the afferent nerves III and IV [54], which stimulate areas of the brain responsible for pain sensation [55]. The findings of Fogaça et al. reported no difference in RPE in post-training between CAF and PLA conditions using the Borg 10-point scale [14]. It has also been shown that the 10-point Likert scale for RPE may not be sensitive enough to record perceptual changes during CrossFit protocols [32]. However, the 10- and 15-point RPE scales showed different results for RPE during the resistance and endurance training protocols with CAF supplementation [56–58]. A recent study by Crawford et al. showed that a 15-point RPE scale might be more appropriate for CrossFit participants [59].

Our results showed that NaHCO_3_ supplementation separately and in combination did not affect the MHR during the Cindy protocol in CrossFit participants (Figure 3c). Contrary to our results, Lorino et al. reported a decrease in heart rate with sub-maximal exercise after consuming 3.3 mg/kg BW of CAF [60]. Also, a decrease of 5 mg/kg of CAF on the heart rate after aerobic activity with an intensity of 30–70% VO_2_ max was observed on a stationary bicycle [61]. Bell et al. showed that consumption of 5 mg/kg BW CAF (even in moderate and high-intensity exercises) led to a significant increase in heart rate after maximal exercise (80–85% VO_2_ max) but does not affect heart rate while riding a bicycle with an intensity of 50% VO_2_ max [62].

Finally, our findings showed that NaHCO_3_ and CAF supplementation combined with NaHCO_3_ caused significant GI side effects compared to the PLA group (Figure 3d). These included minor heartburn and mild diarrhea. The GI side effects observed in the combined condition are probably due to the consumption of NaHCO_3_ because no significant GI side effects were observed in the CAF condition. These complications were temporary and did not impair participants’ performance. Acute consumption of NaHCO_3_ is associated with GI upset [63]. In the present study, to minimize GI discomfort, a gradual loading strategy was adopted 3 days before each assessment session by dividing the daily bicarbonate dose into three equal portions consumed with breakfast, lunch, and dinner. A study by McNaughton et al. suggested that the increase in blood carbonate levels following this loading strategy may be maintained 1 day after consuming 0.5 g/kg BW of SB [24]. Our results confirmed that the loading strategy has a long-lasting effect. Our findings follow Delextrat et al. on female basketball players [64] and Driller et al. (2012) who studied trained cyclists [65], the findings of our study contrasted with those of Durkalec-Michalski et al. (2018), who engaged in competitive crossovers as part of their investigation [9]. Contradictions among these studies are the difference in the dosage of supplementation [66].

Limitations of this study do exist. Before the trial started, participants were well familiarized with the Cindy protocol. Even though the findings were not statistically significant, a practice effect might have impacted them. The small sample size of our study is another limitation. Participants were selected from just one CrossFit gym to ensure uniformity of training regimens throughout the research. More research with a larger sample size may be required to confirm the conclusions of this study. In addition, the primary limitation identified in the present study pertains to the lack of control over extracurricular physical activities that participants engaged in outside the scope of the Koohpaye Box club. Additionally, the variable values of sleep before each intervention session also presented a potential confounding factor. Klier et al. reported a significant positive effect of sleep quality on sports performance in CrossFit participants [67].

## 5. Conclusion

In general, the results of the present study confirm some previous studies and contradict others on the changing power, strength, MHR, RPE, and exercise performance. The study showed that the supplementation of CAF and SB together could not provide further benefits in improving performance and performance-related factors in CrossFit participants. Furthermore, due to the lack of significant changes in athletic performance and MHR, it can be concluded that CAF and SB supplementations, separately and in combination, have the same effect on performance and MHR in CrossFit participants during the Cindy protocol.

## Data Availability

The datasets generated and analyzed during the current study are not publicly available due to ethical restrictions; however, they are available from Amirhosein Ziyaiyan (A.Ziyaiyan@ut.ac.ir) on reasonable request.

## Author details

1. M.Sc., Department of Sports Physiology, Faculty of Physical Education and Sports Science, University of Tehran, Tehran, Iran. 2. Associate Professor, Department of Sports Physiology, Faculty of Physical Education and Sports Sciences, University of Tehran, Tehran, Iran. 3. Ph.D., Department Food and Nutrition, Munich, Consumer Centre of the German Federal State of Bavaria, Germany.

## Abbreviations

CON: Control
PLA: Placebo
CAF: Caffeine
SB: Sodium bicarbonate
BW: Body weight
MP: Muscular power
HGS: Handgrip strength
MHR: Maximum heart rate
H^+^: Hydrogen ion
RPE: Rate of perceived exertion
